# The Breathing, Thinking, Functioning clinical model: a proposal to facilitate evidence-based breathlessness management in chronic respiratory disease

**DOI:** 10.1038/s41533-017-0024-z

**Published:** 2017-04-21

**Authors:** Anna Spathis, Sara Booth, Catherine Moffat, Rhys Hurst, Richella Ryan, Chloe Chin, Julie Burkin

**Affiliations:** 1 Department of Palliative Care, Cambridge University Hospitals NHS Foundation Trust, Cambridge, UK; 2 University of Cambridge, Cambridge, UK

## Abstract

Refractory breathlessness is a highly prevalent and distressing symptom in advanced chronic respiratory disease. Its intensity is not reliably predicted by the severity of lung pathology, with unhelpful emotions and behaviours inadvertently exacerbating and perpetuating the problem. Improved symptom management is possible if clinicians choose appropriate non-pharmacological approaches, but these require engagement and commitment from both patients and clinicians. The Breathing Thinking Functioning clinical model is a proposal, developed from current evidence, that has the potential to facilitate effective symptom control, by providing a rationale and focus for treatment.

## Introduction

Breathlessness is prevalent in chronic respiratory disease, and primary care bears much of the burden of its management. Over 80% of patients in the community with chronic obstructive pulmonary disease (COPD), for example, experience some degree of breathlessness.^1, 2^ It is a debilitating symptom, with a negative impact on quality of life, psychological wellbeing and functional status.^3^ Multiple observational studies link breathlessness with anxiety and depression.^4, 5^ Carers also suffer significantly, burdened by distress, social isolation and a devastating sense of helplessness.^6^

The management of breathlessness is hampered by its inherent complexity. Optimising treatment of the underlying disease has an inconsistent impact on the symptom. There are a plethora of non-pharmacological approaches to ameliorate breathlessness, without evidence to guide individualisation of therapy. Furthermore, understandable, but unhelpful, reactions to the perception of breathlessness unwittingly compound the symptom.

In this paper, we explore this complexity and outline a proposal, developed from current evidence, on how to overcome it using the Breathing, Thinking, Functioning (BTF) clinical model. Although we focus on breathlessness management in COPD, this pragmatic clinical approach can be used in any advanced cardio-respiratory disease.

## Understanding the complexity

### Relationship between disease and breathlessness severity

Breathlessness severity is not reliably predicted by the severity of airflow limitation in COPD. Multiple studies have shown marked variation in the perception of breathlessness, for a given level of lung function.^7, 8^ An observational study of almost 50,000 patients with COPD in the UK revealed that a third of those with stage 1 disease described moderate to severe breathlessness, whereas over 20% of those with stage 4 disease did not have clinically significant breathlessness.^2^ In a recent controlled trial, bronchoscopic lung volume reduction significantly improved FEV1 by 25%, without relief of breathlessness.^9^

The inconsistent relationship between pathology and breathlessness perception explains why optimising disease management alone does not guarantee good symptom control. This discrepancy has led to a number of terms suggesting exaggerated symptom perception, such as ‘disproportionate breathlessness’, ‘breathlessness out of keeping with pathology’ and ‘medically unexplained breathlessness’. It is apparent that there must be factors influencing breathlessness beyond that expected due to the underlying disease.

### Neurophysiology of breathlessness

Neuroimaging studies over the last decade have significantly increased understanding of the neural processing of the sensation of breathlessness, and the range of factors influencing the symptom perception.^10, 11^ Demand for ventilation, sensed by chemo-receptors and metabo-receptors, is compared with afferent information on actual ventilation from widespread sources, including pulmonary stretch receptions, pulmonary C fibres, chest wall joint and skin receptors, and skeletal muscle ergoreceptors.^12^ Breathlessness perception is believed to be driven by a mismatch between the two.^13, 14^ Neural integration occurs in cortico-limbic regions of the brain, which are influenced by thoughts and emotions, further modulating breathlessness perception.^11, 15^

A consequence of this neural complexity is that it limits the potential for a single effective intervention, a point supported by an increasing body of literature (Table 1). Oxygen therapy, although it reverses hypoxaemia, does not improve breathlessness in patients with cancer, and the marginal benefit seen in a meta-analysis of COPD patients is of dubious clinical significance.^16, 17^ Benzodiazepines are widely used, but do not improve breathlessness.^18^ Even opioids, with most evidence to support their use, provide benefit of only borderline clinical significance.^19, 20^

**Table 1:** Summary of key evidence for approaches to the management of breathlessness in advanced disease

Reference	Description	Impact on breathlessness and other key outcomes
*Non pharmacological approaches*		
Bausewein *et al.* 2008^22^	Cochrane systematic review of 47 controlled studies (2532 participants) evaluating non-pharmacological approaches in any advanced disease. Studies evaluating pulmonary rehabilitation, exercise and self-management education excluded	High strength of evidence for neuromuscular electrical stimulation and chest wall vibration
		Moderate strength of evidence for walking aids and breathing retraining
		Low strength of evidence for acupuncture/pressure
Zwerink *et al.* 2014	Cochrane systematic review of 28 controlled studies evaluating self-management interventions in COPD	Significant reduction in breathlessness and respiratory-related hospital admissions
		Improved health-related quality of life
Howard *et al.* 2014^21^	Randomised controlled trial involving 222 COPD patients allocated to receive a cognitive-behavioural manual (applying CBT techniques within a self-management framework, with brief telephone support) or an information booklet	Significant improvement in breathlessness at 6 months (secondary outcome)
		Reduction in A&E visits by 42% with associated cost-savings, improved anxiety, depression
McCarthy *et al.* 2015^24^	Cochrane systematic review of 65 randomised controlled trials evaluating pulmonary rehabilitation in COPD	Moderately large and clinically significant improvement in breathlessness.
		Also improvements in fatigue, quality of life and emotional function
*Pharmacological approaches*		
Abernethy *et al.* 2003^19^	Randomised controlled crossover trial of oral morphine sustained release 20 mg twice daily for 4 days vs. placebo, involving 48 opioid-naive participants	6.6–9.5% improvement in breathlessness More distressing constipation in the opioid group despite laxatives
Abernethy *et al.* 2010^43^	Randomised controlled trial of 239 participants (152 with COPD) with life-limiting illness, 2 l/min oxygen or room air via a concentrator for at least 15 h/day	Improvement in both oxygen and room air group, but no significant differences between groups
Barnes *et al.* 2016^20^	Cochrane systematic review of 26 controlled trials evaluating opioids for refractory breathlessness in any advanced disease	Low quality evidence of improvement in post-treatment breathlessness, but no statistically significant change of breathlessness from baseline
Simon *et al.* 2010^18^	Cochrane systematic review of seven controlled trials evaluating benzodiazepines in advanced malignant or non-malignant disease	No beneficial effect of benzodiazepines, including from a meta-analysis of six of the seven studies
Uronis *et al.* 2008^16, 44^	Systematic review and meta-analysis of five studies evaluating oxygen for dyspnoea in mildly- or non-hypoxaemic cancer patients	Oxygen did not improve breathlessness
Ekstrom *et al.* 2016^17^	Cochrane systematic review of 33 randomised controlled trials evaluating oxygen for dyspnoea in patients with COPD who do not qualify for home oxygen therapy	Oxygen improved breathlessness by the equivalent of 0.7 cm in a 10 cm visual analogues scale, on exercise only.
		There was no benefit from oxygen before exercise, and no improvement in quality of life.
*Complex interventions from specialist breathlessness services*		
Bredin *et al.* 1999^45^	Randomised controlled trial of 119 patients with lung cancer receiving a nurse-led, non-pharmacological, outpatient intervention or best supportive care	Significant improvement in breathlessness, as well as performance status, anxiety and depression, at 8 weeks
Farquhar *et al.* 2014^25^	Randomised controlled trial involving 67 cancer patients, receiving the Breathlessness Intervention Service (multidisciplinary team providing predominantly home-based, non-pharmacological approaches) or usual care	Distress from breathlessness improved significantly (primary outcome) at 2 weeks
		Evidence of cost-effectiveness
Higginson *et al.* 2014^26^	Randomised controlled trial involving 105 patients with mixed advanced disease, receiving Breathlessness Support Service (multidisciplinary team providing predominantly outpatient and home-based, predominantly non-pharmacological approaches) or usual care.	Mastery of breathlessness improved significantly by an average of 16% (primary outcome), at 6 weeks
		Significant improvement in survival in the intervention group
Johnson *et al.* 2015^27^	Randomised controlled trial involving 156 cancer patients receiving either three or a single breathing technique training session	Improvement in worst breathlessness in last 24 h in both groups, but not difference between groups
		The single session was cost-effective

Instead the most successful interventions appear to be multi-modal, non-pharmacological approaches that work concurrently at multiple points within the brain, respiratory and skeletal system.^21–23^ There is strong evidence, for example, that pulmonary rehabilitation, involving exercise, education and support over many weeks, leads to clinically significant reduction in breathlessness, as well as substantial improvements in fatigue, emotional functioning and quality of life.^24^ Approaches incorporating cognitive behavioural therapy ameliorate breathlessness, as well as anxiety and depression.^21^ Evidence is emerging for the effectiveness of breathlessness services, providing a range of predominantly non-pharmacological approaches, such as breathing and relaxation techniques, use of a handheld fan, cognitive behavioural therapy, activity management and carer support. Such services provide a brief, cost-effective intervention that impacts positively on distress from breathlessness, mastery, quality of life, and even survival.^25–27^

### Reactions to breathlessness

There is growing evidence for a further level of complexity: the human reaction to the perception of breathlessness may worsen the symptom. Comroe, an eminent cardiologist, showed great prescience when he wrote in 1966 that breathlessness ‘involves both the perception of the sensation by the patient and his reaction to it…’

Neuroimaging studies reveal that breathlessness activates cortico-limbic areas, particularly the right anterior insula.^10, 11^ These regions subserve awareness of threats such as thirst, hunger, acute pain and breathlessness. Interoceptive awareness of an acute homoeostatic threat leads to an avoidance behaviour motivated by emotion, which, in evolutionary terms, clearly confers a survival advantage. However, in the context of chronic perceived threats such as persistent breathlessness, such emotions and behaviours can be unhelpful and inadvertently perpetuate the problem. For example, attempting to avoid the perception of breathlessness by reducing activity, leads to deconditioning that, in turn, worsens breathlessness. Deconditioning could, in part, explain the evidence in longitudinal studies for progression in breathlessness severity, despite stable lung function.^28^

In a context where optimising management of the underlying disease is insufficient, where no single effective intervention for breathlessness exists, and where patients and clinicians risk being overwhelmed by a myriad of non-pharmacological approaches, crucially, these inadvertently unhelpful reactions provide a valuable opportunity to intervene.

## The BTF clinical model

**Fig. 1:**
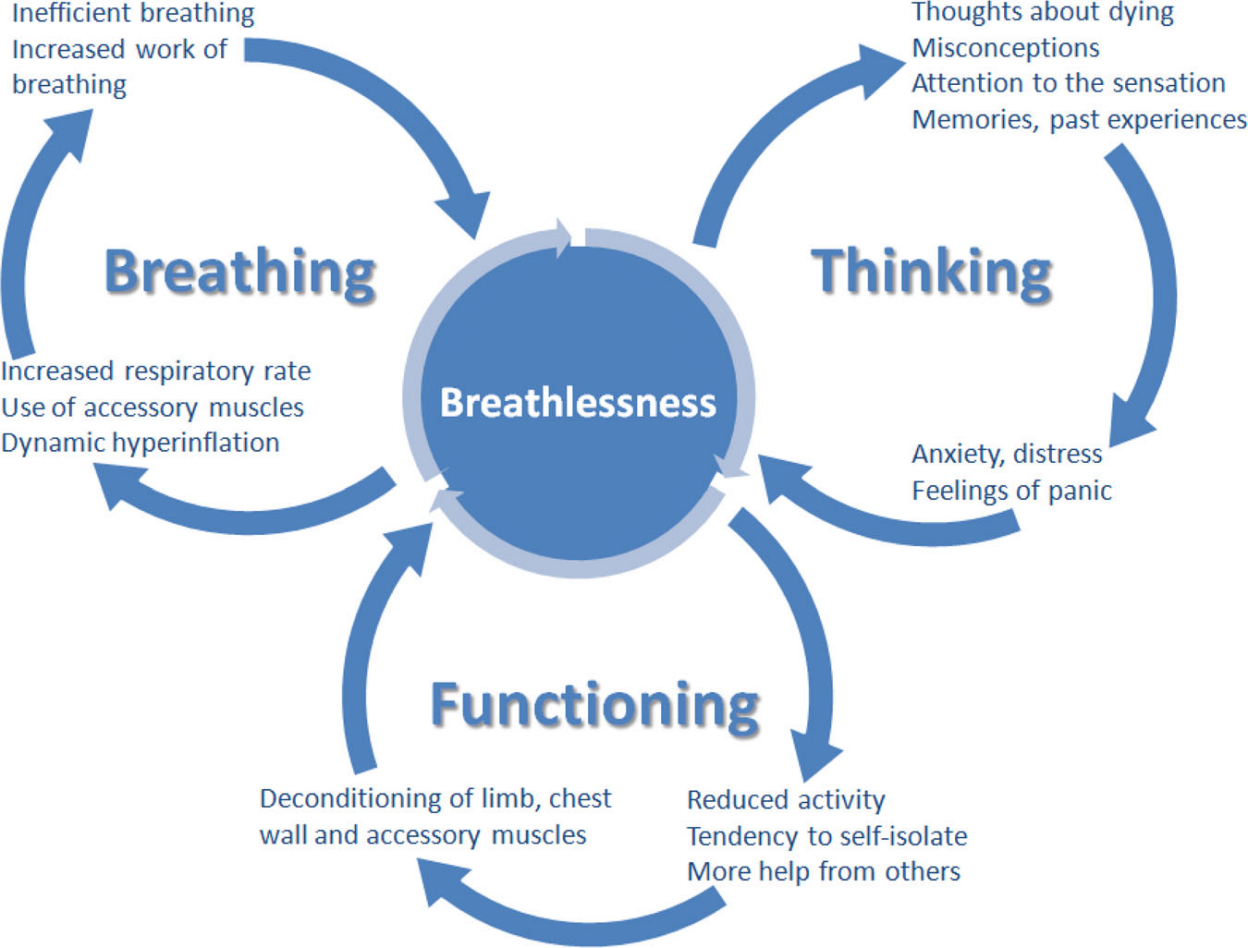
The Breathing, Thinking, Functioning clinical model

The model is based on three predominant cognitive and behavioural reactions to breathlessness that, by causing vicious cycles, worsen and maintain the symptom (Fig. 1).

### Breathing domain

Dysfunctional breathing patterns are well recognised in breathless individuals without underlying respiratory pathology.^29^ Features include apical breathing, higher ratio of inspiratory to expiratory length, absence of end-expiratory pause and frequent sighs or yawns.^30^ Breathing pattern disorders occur in about a third of asthma patients,^31^ and although there has been no research in other respiratory conditions, such as COPD, there is extensive anecdotal evidence that dysfunctional breathing patterns do occur. Breathless patients experience a sense of ‘needing more air’ and, therefore, may consciously or subconsciously increase their tidal volume or respiratory rate, breathing predominantly using the upper chest and accessory muscles. Apical breathing causes reliance on fatiguable accessory muscles of respiration, underutilising the efficient and relatively fatigue-resistant diaphragmatic muscle, further increasing the work of breathing and intensifying breathlessness.^32^

### Thinking domain

It is well-recognised that the anxiety or fear caused by breathlessness can, in turn, augment the perception of breathlessness.^33^ This vicious cycle can lead to a rapidly escalating sense of panic, which most breathless patients experience at some point. The neural processing for this feedback loop is likely to occur in the cortico-limbic areas of the brain, involved in both breathlessness perception and processing of emotion.^34^ In addition, anxiety increases the respiratory rate and can cause muscle tension in both the ventilatory pump and other skeletal muscles, so further increasing the work of breathing and respiratory demand.

### Functioning domain

As breathlessness is so unpleasant, it is natural to avoid it by reducing activity. However, inactivity leads to muscle deconditioning, with reduced oxidative capacity, muscle fibre atrophy and fibre type shift from type 1 (oxidative) to type IIb (glycolytic) fibres.^35^ This increases the demand on the respiratory system, and worsens breathlessness further. Patients intuitively understand this ‘deconditioning vicious cycle’, being aware that less ‘fit’ people are more breathless on exertion. Family members and other carers can unwittingly compound the situation, by trying to help through doing activities that the patient might otherwise have done.

### Model development

This empirical model was developed by the Cambridge Breathlessness Intervention Service (CBIS), a multidisciplinary home-based intervention of proven cost-effectiveness.^25^ Each of the three domains is supported by research evidence, as described above, and the model is theoretically underpinned by a ‘vicious flower’ maintenance model formulation, widely used in cognitive–behavioural therapy within psychological medicine.^36^

It was initially conceived in 2012 for use as an educational tool for healthcare staff, to explain mechanisms worsening breathlessness beyond the underlying respiratory condition, and to facilitate choice of non-pharmacological techniques to interrupt each vicious cycle. As well as being enthusiastically endorsed by healthcarers for providing a conceptually clear approach to the management of a complex symptom, service evaluations and anecdotal evidence from clinical utilisation within CBIS are revealing unanticipated benefits for patients and their families.

## Potential roles in supporting self-management in primary care

### Explaining and engaging

The evidence is clear that when individuals with a chronic condition, like COPD, actively participate in their own care through self-management, their medical outcomes improve.^37, 38^ Non-pharmacological interventions are under-utilised, not least because patients need commitment to make and sustain behaviour change. Providing a clear and logical rationale for symptom deterioration, or perpetuation beyond an initial trigger, can be profoundly motivating.

Patients, furthermore intuitively comprehend that a vicious cycle can be broken, turning instead into a ‘cycle of improvement’. The ‘small changes approach’, accepted as a particularly effective and feasible way of achieving behavioural change, develops the potential to generate rapid and large gains.^39^ Enhancing self-efficacy is a particularly effective way to support self-management.^40^

Engaging healthcare professionals is equally vital. Clinical training prioritises disease-modification over symptom control and, unlike allied health professionals, doctors are rarely taught how to support behaviour change. The focus is on prescribing, a relatively simple, culturally acceptable and passive practice compared to the time, energy and cohesive teamwork required to deliver non-pharmacological therapies. Providing a rationale, not just for the presence of the symptom, but also for why it should be amenable to intervention, can be helpful for clinicians too.

### Facilitating and focusing management

**Fig. 2:**
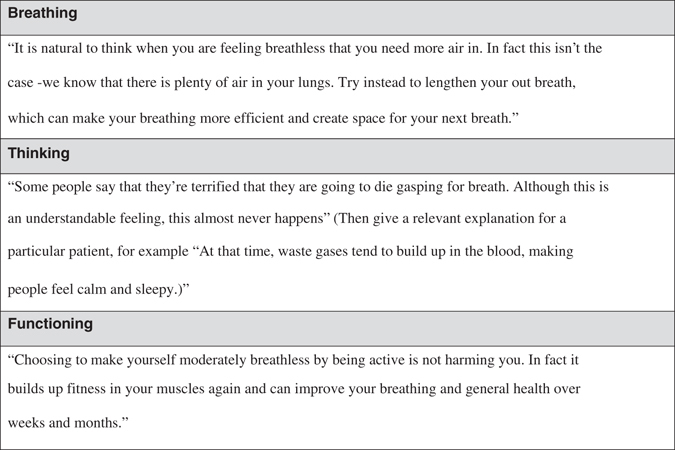
Challenging the misconceptions driving each vicious cycle

Patients with intractable breathlessness are commonly fatigued, which limits the capacity to retain and process new information.^41^ The more information presented in a consultation, the lower the proportion that is correctly recalled.^42^ The wide array of potentially effective non-pharmacological interventions for breathlessness fit neatly within the three domains of the clinical model (Table 2). Clinical experience shows that, although all three vicious cycles occur to a degree in each breathless patient, in practice, one or two tend to predominate. Focusing on those interventions which impact on the predominant cycle can increase adherence and, importantly, provide early ‘quick wins’ that further contribute to patient engagement and self-efficacy. Simply, for example, compassionately challenging one of the misconceptions driving a predominant vicious cycle (Fig. 2) can, in itself, be highly effective and time-efficient in a busy healthcare setting.

**Table 2:** Categorisation of symptom management approaches according to Breathing, Thinking, Functioning domain

Breathing	Thinking	Functioning
Breathing techniques	Cognitive behavioural therapy	Pulmonary rehabilitation
Handheld fan	Relaxation techniques	Activity promotion
Airway clearance techniques	Mindfulness	Walking aids
Inspiratory muscle training	Acupuncture	Pacing
Chest wall vibration		Neuromuscular electrical stimulation
Non-invasive ventilation

## Next steps

Although, as described, the model underpins the delivery of an evidence-based intervention and each of the three domains are supported by research evidence, formal research evaluation of the model is required. A mixed-methods approach is planned. Phenomenological qualitative research methods, with in-depth interviews and focus groups, will gather and triangulate insights from patients, carers and healthcare staff, in terms of experiences of using the model and attributed meaning. As anecdotal evidence suggests that the model could have a particular role in facilitating brief community-based intervention, a cluster-randomised trial will be undertaken to evaluate the cost-effectiveness of a single intervention for patients with COPD in primary care. It will initially be tested in patients with severe disease who remain symptomatic after, or could not complete, pulmonary rehabilitation, and those considered, but ineligible, for invasive procedures like lung volume reduction or transplant. Key outcomes will include changes in perceived self-efficacy in symptom management, in the rate of primary care consultations and in the number of contacts with emergency services out of hours.

## Conclusion

Insight into the many factors influencing breathlessness in chronic respiratory disease, including the inadvertently unhelpful reactions that exacerbate it, provide a valuable opportunity to intervene.

We propose that the BTF model not only explains how breathlessness perpetuates and worsens, but also provides a structure and rationale for its management—through generating the antithesis of vicious cycles, ‘cycles of improvement’—thereby engaging patients and professionals alike. Given the availability of diverse non-pharmacological approaches, the model potentially provides direction and focus to initial management, facilitating the provision of personalised therapy.

Approaches that promote self-management are known to benefit people with chronic conditions. Time-pressured clinical settings require a brief intervention that targets the specific mechanisms driving an individual’s symptoms. The BTF model has the potential to fulfil all these needs, and may be particularly relevant in community settings. Further research is required to clarify its effectiveness.
